# Investigating the free-roaming dog population and gastrointestinal parasite diversity in Tulúm, México

**DOI:** 10.1371/journal.pone.0276880

**Published:** 2022-10-27

**Authors:** Michael A. Lyons, Rumaan Malhotra, Cody W. Thompson

**Affiliations:** 1 Department of Ecology and Evolutionary Biology, University of Michigan, Ann Arbor, Michigan, United States of America; 2 Museum of Zoology, University of Michigan, Ann Arbor, Michigan, United States of America; University of Lincoln - Brayford Campus: University of Lincoln, UNITED KINGDOM

## Abstract

Free-roaming domestic dogs (*Canis lupus familiaris*) pose major conservation and public health risks worldwide. To better understand the threat of domestic dogs to wildlife and people and add to the growing literature on free-roaming dog ecology, a study was conducted to estimate the dog population in Tulúm, México. A modified mark-recapture technique and program MARK were used to obtain dog population estimates along six different transects dividing the city. Population estimates ranged from 19.75 dogs in one transect to 101.841 dogs in another, with 150 total dogs identified throughout the study and an estimated minimum population density of 48.57 dogs/km^2^. Fecal samples were also opportunistically collected for parasite identification through fecal flotation analysis using the McMaster technique. Out of 25 samples collected, 19 tested positive for gastrointestinal parasites with the most common species found being *Ancylostoma caninum*, followed by *Toxocara canis*, *Dipylidium caninum*, and *Cystoisospora* spp. Parasite loads ranged from 50 to 10,700 ova per gram of feces. The large population of free-roaming dogs and the prevalence of three zoonotic parasites highlight the importance of understanding free-roaming dog ecology and educating the public on the health risks free-roaming dogs pose. Los perros callejeros *(Canis lupus familiaris)* representan un gran riesgo para la conservación de animales y la salud pública mundialmente. Para comprender mejor la amenaza que significan los perros domésticos para la fauna silvestre y los humanos y aportar a la creciente bibliografía sobre la ecología de los perros callejeros, se realizó una investigación para estimar la población de los perros en Tulúm, México. Se utilizó una técnica modificada de marcado y recaptura junto con el programa MARK para estimar la población canina en seis transectos de la ciudad. Los estimados varían desde 19.75 perros en un transecto hasta 101,841 en otro, con un total de 150 perros identificados en el transcurso de la investigación y una densidad mínima estimada de 48,57 perros/km^2^. Además, se hizo una recolección oportunista de muestras de heces para la identificación de parásitos por medio del análisis de flotacíon fecal, con el método McMaster. De las 25 muestras recolectadas, 19 resultaron positivas para parásitos gastrointestinales, de las cuales las especies más comunes fueron *Ancylostomoa caninum*, seguida por *Toxocara canis*, *Dipylidium caninum*, y *Cystoisospora* spp. Las cargas parasitarias variaron desde 50 hasta 10.700 óvulos por gramo de heces. La alta población de perros callejeros y la prevalencia de tres enfermedades zoonóticas resaltan la importancia de entender la ecología de los perros callejeros y educar al público sobre los riesgos que significan los perros callejeros para la salud.

## Introduction

Domestic dogs (*Canis lupus familiaris*) were domesticated from gray wolves (*C*. *lupus*) between 20,000 and 40,000 years ago [1]. Since their domestication, people have brought domestic dogs around the world, to regions far beyond the range of their wild counterparts. Beyond contributing to the spread of domestic dogs, the association between dogs and people have allowed domestic dog populations to grow dramatically to an estimated worldwide population of 900 million, far beyond the population size of any wild carnivore [2]. These 900 million dogs fall into two main categories: 1) confined dogs and 2) free-roaming dogs [3]. Free-roaming dogs include a combination of feral, stray, and owned dogs that are allowed to roam outside without human supervision. An estimated 70 to 75% of the worldwide dog population is free roaming [3]. Cultural variation in the treatment of dogs impacts whether dogs are allowed to roam freely or are confined, leading to geographic variation in the concentration of free-roaming dogs [4]. In México, the dog population is estimated to be around 19.5 million dogs, 70% of which are free-roaming dogs [5].

Large populations of free-roaming dogs can greatly influence wildlife through a variety of interactions [6], affecting the persistence and abundance of carnivore populations [7–9]. Free-roaming dogs also predate on native wildlife, sometimes threatening endangered species [10]. In protected areas around México City, free-roaming dog activity had a negative impact on medium-sized wild mammal species (e.g., fox, opossum, raccoon, weasel) richness, activity, and abundance, indicating that free-roaming dog populations could be hindering the coexistence of humans and wildlife in urban areas around México [11].

Additionally, free-roaming dogs have the potential to impact wildlife populations through disease transmission, being implicated as the causes of rabies and canine distemper outbreaks [4,12,13]. Free-roaming dogs in México have been identified as a potential threat to carnivore populations through the transmission of the canine distemper virus in the Janos Biosphere Reserve and the transmission of parvovirus and toxoplasmosis to wildlife species in México City [14,15]. The role free-roaming dogs play in disease transmission can have large impacts on wildlife populations with dog-caused rabies and distemper outbreaks sometimes leading to reductions in wildlife populations [16–18].

In addition to wildlife, free-roaming dogs pose a similar threat to human health through disease transmission. Dogs and humans share over 60 parasite species, including *Giardia*, hookworms, and tapeworms, meaning dogs could play a role in infecting humans with these parasites [19]. The close proximity in which dogs and humans live creates an ideal situation for parasite transmission from dogs to people [20,21]. In addition to being involved in parasite transmission, dogs can spread numerous viruses and bacteria to humans, including rabies, noroviruses, and *Salmonella* species [22]. For example, Jimenez-Coello et al. [23] found that free-roaming dog populations located in Chiapas, México, serve as a reservoir for several pathogens, including *Leptospira interrogans*, *Trypanosoma cruzi*, and *Aspergillus* spp. An additional study analyzing scat found in public parks throughout Campeche, México, found a high prevalence of *Ancylostoma caninum*, a zoonotic gastrointestinal parasite [24]. While these studies are important, more studies like these are still needed to better understand the prevalence of free-roaming dog parasites throughout México and the world, to determine the factors impacting parasite prevalence, and to identify populations at risk for zoonotic infections. Additionally, given that dog population size can be used as a baseline to evaluate the threat of zoonotic infection, more studies that look at population size in conjunction with parasite prevalence are important.

The goal of this study is to estimate the dog population in Tulúm, México, and understand the prevalence and diversity of gastrointestinal parasites from fecal samples found in the city, with special interest in how the free-roaming dog population and parasite community varies throughout the city. We hypothesize that the free-roaming dog population will be higher in areas outside of centers of local tourism. Tulúm is one of the most highly visited cities in México because of the adjacent Zonas Arquelógica de Tulúm, which receives nearly one million visitors annually (Secretaría de Turismo, México). Additionally, we hypothesize that gastrointestinal parasite prevalence will be higher in areas with greater dog densities. When controlling for differences in dog densities, we also hypothesize that parasite prevalence will be higher in lower income areas due to lower use of de-worming medication and in areas closer to forests due to increased contact with wildlife.

## Materials and methods

### Study site & sampling

The study was conducted in Tulúm, Quintana Roo, México from 6 February to 8 April 2021. Tulúm is a popular tourist destination in southern México with a local population of 33,374 people with a transient population that may not be counted in the census [25]. Observational data was collected on individual dogs living in the city of Tulúm to determine population size, and opportunistic scat samples were collected to identify gastrointestinal parasite loads. Data collection for estimating the free-roaming dog population ran from 6 February to 3 March 2021, and scat collection ran from 15 March to 8 April 2021. Given this study was an observational study on public property, no permits or institutional animal care and use protocols were required.

### Modified mark-recapture methods

**Fig 1:**
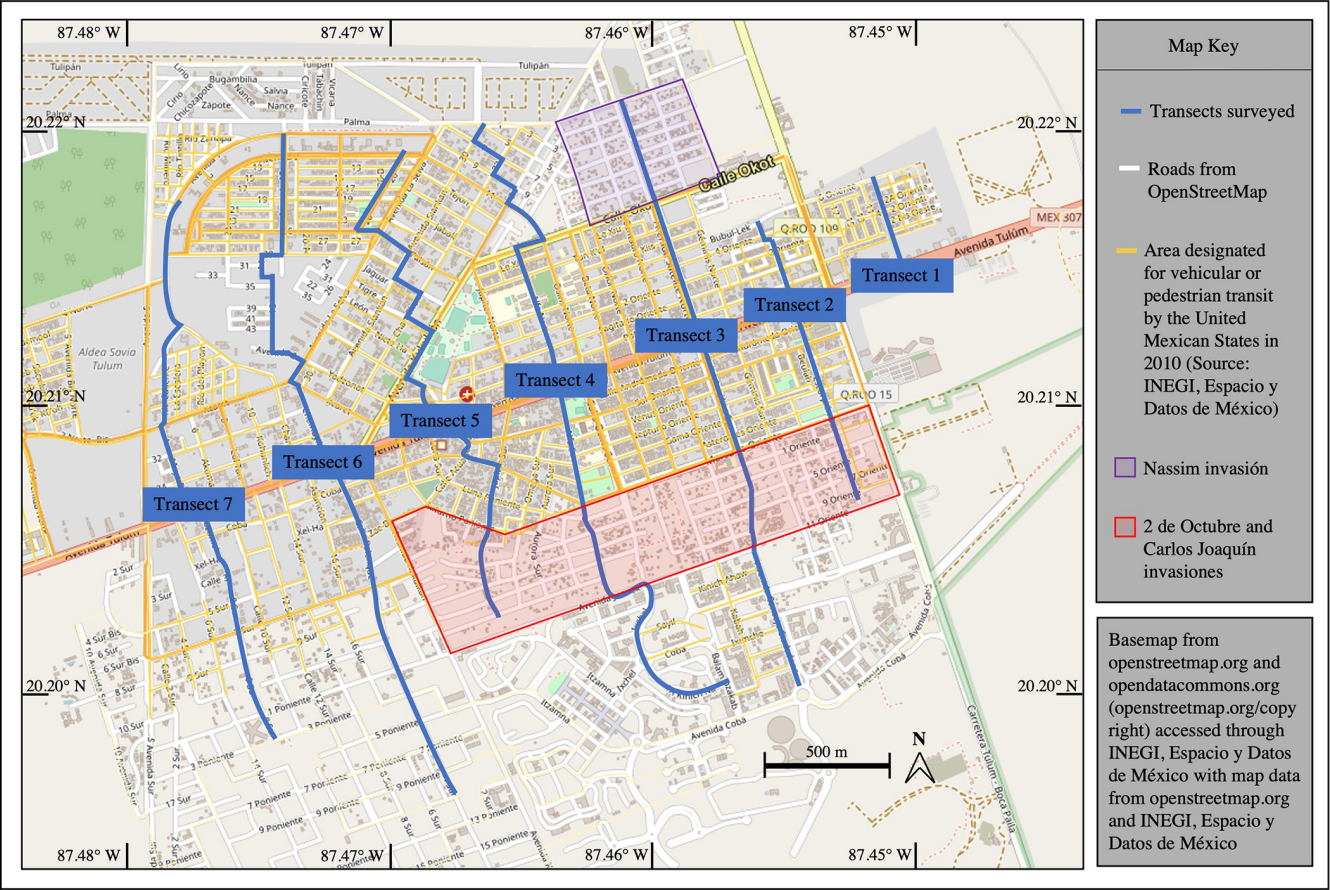
Map of transects sampled in Tulúm, Quintana Roo, México. Transects were used to determine the population status of free-roaming dogs within the city limits.

To estimate the population of free-roaming dogs in Tulúm, México, a modified mark-capture-recapture technique was implemented. Instead of capturing and physically marking individuals, a non-invasive approach using digital photography was used to photograph dogs with a Samsung Galaxy S9 phone camera to identify individuals and match “recapture” events. Seven transects approximately 475 meters apart were plotted across the city of Tulúm (Fig 1). Transects ranged in length from 370 meters to 2,949 meters (see Table 1). Everyday three transects were chosen randomly to be surveyed.

**Table 1:** Description of transects sampled in Tulúm, Quintana Roo, México.

Transect	Length (meters)	AM Trap Days	PM Trap Days	Total Dogs Seen	No. of Encounters	No. of Recaptures	Lincoln-Petersen Population Estimate
1	370	4	6	1	1	0	N/A
2	1142	4	4	15	20	5	26.7
3	2440	4	4	32	49	17	49.9
4	2915	4	4	34^a^	44	10	72
5	2440	4	4	32^a^	48	16	45.5
6	2949	4	6	25	42	17	36.4
7	2390	4	4	12	18	6	18.7
Total =	N/A	28	32	150^a^	222	71	

The length of transects as measured from ArcGIS (ESRI, West Redlands, California) is listed as well as the number of AM and PM trap days, the number of dogs seen on each transect, the number of dog encounters on each transect (i.e., picture of each dog per sampling event), the number of recaptured/resighted dogs, and the Lincoln-Petersen population estimates for each transect.

^a^Since there was one dog found in both transects 4 and 5, total dogs encountered throughout the study is 150, not 151 as adding all values in the final column would imply.

To “catch” dogs at different times of day, transects were biked around 5:30 PM on Mondays, Wednesdays, Fridays, and Sundays, and around 9 AM Tuesdays, Thursdays, and Saturdays until each transect was surveyed four times in the morning and four times in the evening. Since biking three transects per day took around 2 hours total, the order that the randomly selected transects were surveyed was modified daily so that individual transects were not always sampled at the beginning or end of the 2-hour sampling period. For each transect, photographs were taken of every dog encountered unaccompanied by an owner. When possible, multiple photos of single encounters were taken to aid in dog identification. Each photo was timestamped and included geographic coordinates. Sex was recorded when it was easily determined, and notes were taken on whether individuals were seen previously.

### Individual dog identification

**Fig 2:**
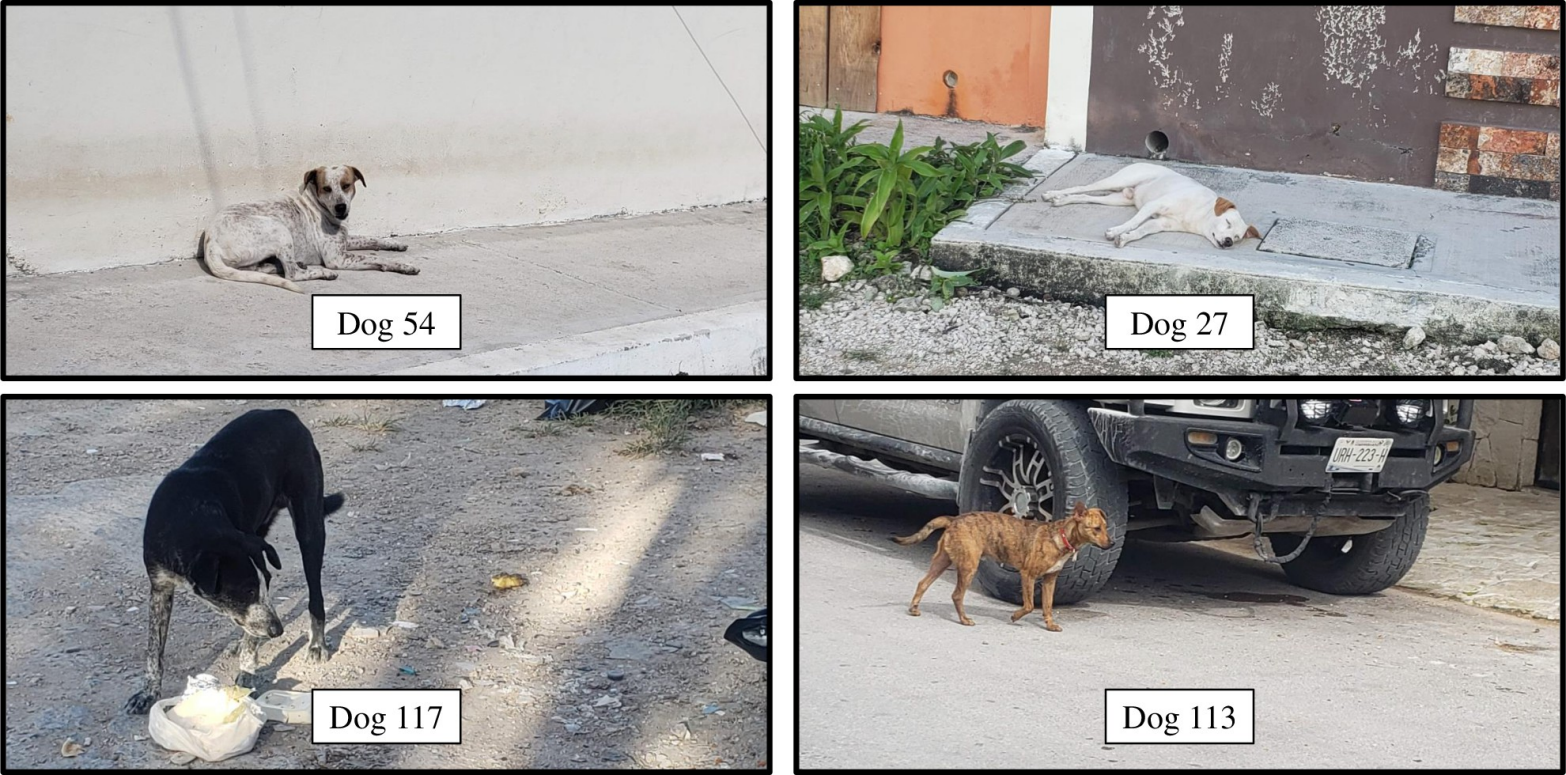
Four dogs from Tulúm, individually distinguishable by phenotypic variation. The top two panels show a pair of individual dogs with similar features but are distinguishable by the extent of brown coloration on their faces. The bottom two panels show two dogs that are clearly different individuals.

Every dog encounter, defined as a picture being taken of a dog, was given a unique collection number. For example, in a sequence of two pictures, one with one dog and the other with two dogs, there would be one collection number corresponding to the first photo and two more unique collection numbers corresponding to the second photo. As each photo was processed, collection numbers were given to each dog encounter, and the date, time, latitude, longitude, transect number, sex, and dog color were recorded. Additionally, each dog was given a unique number as an identifier for the individual starting at number one. Dogs were identified using a combination of traits, such as sex, coat color and pattern, tail size and color, and collar type and color when present. Fig 2 shows a sample of four dogs captured during the study to demonstrate phenotypic differences within the population. To ensure the same individual was not identified as two different dogs, every dog in every photo was compared to all dogs with the same color in previously processed images, all previously encountered dogs on the same transect, and all previously encountered dogs on adjacent transects.

### Lincoln-Petersen population estimates

Using the methods outlined by Menkens and Anderson [26] for using multiple sampling events in the Lincoln-Petersen equation for estimating population size, a baseline estimate of the population size in each transect was found except for Transect 1 since there were no recapture events. In the Lincoln-Petersen equation as presented by Menkens and Anderson [26], $N=\frac{M*S}{R}$, N represents the population estimate; M is the number of marked individuals from the first sampling period; S is the total number of individuals caught in the second sampling period; and R is the number of marked animals from the first sampling period recaptured during the second sampling period. Here, the number of marked individuals from the first sampling period was calculated from the number of unique dogs identified in the first half of sampling days (4 or 5 days depending on the transect). S was then the number of dogs identified in the last 4 or 5 sampling days and R, the number of those dogs which had been previously identified in the first half of sampling days. While the Lincoln-Petersen method is informative for estimating population size, it does not consider survival probability, recapture probability, or time between sampling events.

### Population size, survival probability, and recapture probability

Given the month-long duration of the study, an open population model was needed as individuals can die, immigrate, or emigrate within this time. Along with being an open population model, the POPAN formulation in the program MARK gives additional information about the population that the Lincoln-Petersen estimates do not [27]. First, the total number of individual dogs encountered was determined by identifying images using a three-step process. This estimate could be thought of as the minimum number of dogs in a transect. In order to estimate the number of dogs using POPAN, encounter histories for each individual were compiled. These encounter histories were then input into program MARK and the POPAN formulation was used to estimate population size (*N*), survival or persistence (*phi*), recapture probability (*p*), and entrance probability (*pent*) for each transect except for Transect 1, again due to the lack of recapture events [27,28]. Note that while *phi* will be referred to as survival, it is more useful to think of *phi* as the probability of the dog persisting on the streets, which includes the probability that a dog survives, does not migrate out of the area, and was not later confined by owners or rescuers. Eight different models were run corresponding to every possibility of survival, recapture probability, and entrance probability varying by time or remaining constant throughout the study for each transect (Table 2). Whether a parameter varied by time is denoted in the model notation by a “t” or a “.” indicating the parameter varied by time or remained constant in the model, respectively. Models were accepted based on AICc weight and weighted averages were obtained for unconfounded parameters when more than one model was accepted.

**Table 2:** List of all possible models for analysis for each transect with POPAN in MARK.

Model No.	Model Notation	Number of parameters (8 visits vs. 10 visits)
1	*phi*(t) *p*(t) *pent*(t)	18 or 24
2	*phi*(t) *p*(t) *pent*(.)	14 or 18
3	*phi*(t) *p*(.) *pent*(t)	13 or 17
4	*phi*(.) *p*(t) *pent*(t)	13 or 17
5	*phi*(t) *p*(.) *pent*(.)	9 or 11
6	*phi*(.) *p*(t) *pent*(.)	9 or 11
7	*phi*(.) *p*(.) *pent*(t)	8 or 10
8	*phi*(.) *p*(.) *pent* (.)	4

A “t” corresponds to a parameter varying over time, meaning that models with *phi*(t), for example, had separate *phi* (survival) parameters between each visit to the transect. A “.” indicates that the given parameter was assumed to stay constant throughout the study period and thus for a model with *phi*(.), there would only be one *phi* parameter and thus only one survival rate estimated for the whole duration of the study. The two values listed for number of parameters correspond to when there were 8 visits to a transect vs. 10 visits.

The POPAN formulation assumes that marked and unmarked animals have the same probability of capture/recapture and survival, tags or markings are permanent, markings are not misread, sampling and release of captured animals happens immediately, and the study area is constant throughout the study [27]. Given that no physical markings or tags were used and only pictures were taken as opposed to physically capturing individuals, the survival probability and catchability should not differ between “marked” and “unmarked” individuals and sampling and release of captured animals happened instantaneously. All markings were permanent as unchanging phenotypic characteristics (coat color, sex, etc.) were used to identify individuals and due to high phenotypic diversity in the population and rigorous identification methods misreading “markings” is highly unlikely. Finally, the study area did not change throughout the study. While it’s reasonable to conclude these assumptions were met based on study design, goodness of fit testing can also be used to verify that the data met the expectations under these assumptions. For each transect, the most general model {*phi*(t)*p*(t)*pent*(t)}, where the *phi*, *pent*, *and p* parameters all varied by time, was tested for goodness of fit using tests 2 and 3 from program RELEASE within MARK. Outputs from tests 2 and 3, which test for the assumptions that marked and unmarked animals have the same capture and survival probabilities [29], were used to determine goodness of fit, with p-values under 0.05 indicating a significant lack of fit.

### Scat collection & analysis

Scat was collected opportunistically in two ways between 3 March and 8 April 2021. First, whenever scat was encountered on the road or sidewalk, it was collected. When scat was collected, the date and location of collection was recorded. Scat was also acquired from a local veterinary clinic, Alma Animal Vet Clinic and Rescue, who offered members of the public free fecal flotations for anyone that brought in a stool sample from their dogs. After the results of the fecal flotations were returned to the owners, the ongoing research project was explained to them, and they were given the option of allowing the results of the fecal flotation to be used in the project. All fecal samples were kept in a fridge and processed within 24 hours.

To investigate fecal parasite loads, a modified McMaster fecal flotation technique was used [30]. The following steps were followed using salt water (3/4 cup salt with no additives to 16 ounces of water) as the flotation solution. For each fecal sample, 2 grams of the sample was weighed out and put in an 8-ounce plastic cup. The feces were then crushed using a sterilized plastic spoon and 28 ml of flotation solution was slowly added to the same cup to soften the sample. Once a uniform consistency was achieved and all the flotation solution was added, the fecal sample solution was stirred 30 times. After stirring, the fecal sample solution was poured into a new 8-ounce plastic cup covered with one layer of sterile gauze. The solution was left to strain through the gauze for 3 minutes. Feces caught in the gauze were then discarded. The strained fecal sample solution was then stirred 30 times, at which point the solution was drawn up using a syringe from the top of the mixture. Using the syringe, the two McMaster chambers were filled. The McMaster slide was placed on the microscope and then allowed to sit for five minutes to allow the eggs to float to the top near the gridlines on the slide. Parasite eggs were counted and recorded from both chambers. A veterinarian confirmed the species identifications for each fecal sample. The total number of eggs counted per parasite species identified was then multiplied by 50 to get the number of eggs per gram in the fecal sample.

## Results

### Raw data

A total of 292 photos were taken. 228 images had one dog, 47 images had two dogs, nine images had three dogs, three images had four dogs, two images had five dogs, and two images had six dogs, resulting in a total of 222 dog encounters (Table 1). Seven additional dog encounters did not have images, but they were recorded with voice and hand-written notes. All images for dog observations and the corresponding metadata are deposited at the University of Michigan’s Deep Blue Data repository (https://doi.org/10.7302/yncp-6w10).

Lincoln-Petersen population estimates were found for Transects 2–7 (Table 1). Estimating the population using this method for Transect 1 was not possible since there were no recaptures, leading to a denominator of 0 in the Lincoln-Petersen equation. Presence and absence data for each transect, along with the variables used in the Lincoln-Peterson estimates, are found in the supplementary information (S1 Dataset).

### MARK analysis

For Transects 2–7, only some models (between 4 and 6) were able to be fitted as a result of numerical convergence never being reached. In all transects, all models where the pent parameter varied by time were able to be fitted, indicating that probability of entry does in fact vary over time. Details on what models were able to be fitted for each transect along with their AICc weights can be found in S1–S6 Tables. For each transect the most general model {phi(t)p(t)pent(t)} was tested for goodness of fit using program RELEASE. In all transects the goodness of fit tests indicated good model fit. Weighted averages for each parameter were calculated for Transect 7, while in Transects 2–6 the only model with a significant AICc weight was {phi(.)p(.)pent(t)}. Population size, survival rates, and recapture probability estimates for each transect can be found in Table 3. Note that estimates for the population size of Transect 1 were not found due to the lack of recapture events. Given that only one dog was seen in Transect 1 throughout the study period, the dog population in Transect 1 was considered negligible.

**Table 3:** Results from POPAN analysis for Transects 2–7.

Transect No.	*phi*	95% CI *phi*	*p*	95% CI *p*	*N*	95% CI Pop. Size	Dog population per km
2	0.73	0.55–0.87	0.65	0.09–0.97	19.75	15.51–59.58	17.29 dogs/km
3	0.97	0.79–1.00	0.19	0.10–0.33	60.98	43.82–103.05	24.99 dogs/km
4	0.95	0.89–0.98	0.12	0.06–0.21	101.84	62.87–193.39	34.94 dogs/km
5	0.95	0.82–0.99	0.18	0.09–0.32	65.38	46.14–110.81	26.79 dogs/km
6	0.87	0.76–0.93	0.34	0.20–0.52	38.97	30.43–60.91	13.21 dogs/km
7	0.95	0.69–0.99	0.22	0.07–0.52	23.45	14.71–60.88	9.81 dogs/km
Total =	N/A	N/A	N/A	N/A	310.37	N/A	N/A

Estimated values and 95% confidence intervals for *phi* (survival probability), *p* (capture probability), and N (population size) for transects 2–7. Weighted averages for each value were calculated for Transect 7. The last column shows the estimated dog population size for each transect divided by the transect length in kilometers. These values serve to control for variation in transect length when comparing the dog population size between transects.

**Fig 3:**
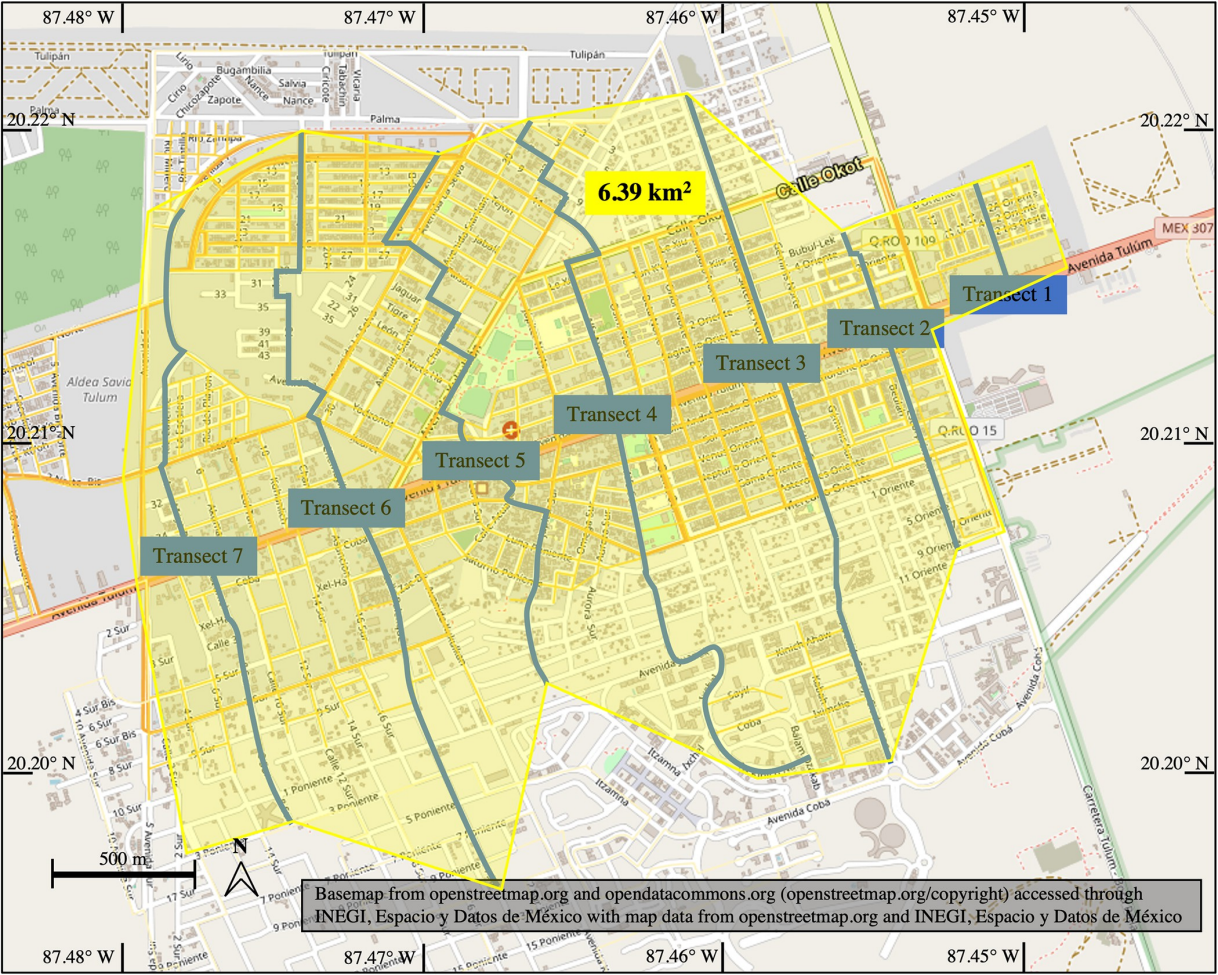
Map of Tulúm with the sampled area outlined. The sampled transects are indicated.

Adding the dog population estimates for each transect gives a total of 310 dogs (see Table 3). The total area of the city sampled, defined as the area extending from around 237.5 m to the west of Transect 7 to 237.5 m to the east of Transect 1, is 6.39 km^2^ (Fig 3). This leads to a minimum dog population density estimate of 48.57 dogs/km^2^. However, considering that only one dog was seen in two transects, it is likely that dogs living between transects were not counted and the total dog population estimate is low.

Survival rates were generally high among different transects while capture probabilities were low. Transects 3, 4, 5, and 7 had survival probabilities of 95% or higher, while Transect 6 had an estimated survival probability of 87% and Transect 2 had an estimated survival probability of 73%. On the other hand, capture probabilities for Transects 3–7 were below 35%. Transect 2 did not fit this trend, with a higher recapture probability of 65%. Most differences in survival and capture probabilities between transects were insignificant under a 95% confidence interval, showing overlap between confidence intervals. Only the survival probabilities between Transect 2 and 4 were significantly different with Transect 2 having a lower survival probability than Transect 4.

While the dog population estimates for each transect provides a basis to compare different transects, it is important to remember that the transects were not all the same length (Table 1). To get a better understanding of how the dog population varied throughout the city, the estimated dog population for each transect, as reported in Table 3, was divided by the transect length to control for variation in length between transects (Table 3). The order of transects in terms of largest population size remained the same when controlling for length of transect, except for Transect 2 which had a larger number of dogs per kilometer of transect than Transects 6 and 7 but a lower overall population size.

### Parasite loads

Of 25 scat samples collected, 19 harbored parasite eggs (76%). Four different parasite species were identified: *Ancylostoma caninum*, *Cystoisospora* spp., *Dipylidium caninum*, and *Toxocara canis*. The most common parasite identified was *A*. *caninum*, which was present in 16 scat samples (64% of all scat samples collected and 84.2% of scat samples harboring parasite eggs). Parasite loads ranged from 50 *A*. *caninum* eggs per gram of feces to 10,700 eggs per gram with an average of 1781.25 eggs per gram (EPG). *T*. *canis* ova were found in two scat samples that also had *A*. *caninum* ova. Parasite loads were 50 and 350 EPG. *Cystoisospora* ova were also found in two scat samples with parasite loads of 50 and 2450 EPG. Finally, *D*. *caninum* was only found in 1 scat sample with a load of 600 EPG.

## Discussion

Understanding domestic dog population demographics is useful for understanding the threats they pose to wildlife and humans, as information on dog densities and population sizes can be used in conjunction with information from other areas to make better estimates of country-wide and worldwide free-roaming dog populations [4]. Estimates of domestic dog densities and population sizes can be used to determine the potential for contact with human and wildlife populations and can thus impact the spread of disease. A lack of studies addressing local dog populations and densities has made comparing domestic dog population demographics and understanding the reasons behind variation in dog population densities difficult [4]. Our study adds to the growing literature on free-roaming dog population demographics.

### Tulúm free-roaming dog population

As noted above, the initial population density estimate for Tulúm was 48.57 dogs/km^2^, but the fact that only one dog was found in more than one transect suggests that this is an underestimate. The literature on free-roaming dogs reports home range sizes in urban areas of 0.037 km^2^ in Panchkula, India [31], 0.057 km^2^ in Guatemala and Uganda, and 0.056 km^2^ in Indonesia [32]. With the lower estimate of home range size, a dog could live in between transects set 0.475 km apart without ever crossing a transect, which is compatible with the finding that only one dog crossed two transects in this study. The lack of overlap in individuals between transects indicates that home ranges sizes are likely smaller than the distance between transects, meaning our values for dog population and density are likely underestimates. It is important to point out that in many areas around the world domestic dog home range sizes are much larger, bringing up important questions of why home range size seems to be smaller in Tulúm and what factors impact home range size. Previous studies on the relationship between free-roaming domestic dog home range sizes and food availability have found that dogs living in resource rich areas tend to have smaller home range sizes [33] and dogs given more supplemental food by their owners had smaller home range sizes [34]. The lack of dogs moving between transects could be a result of high food availability, whether human provided or scavenged/hunted, in Tulúm.

Previous studies have found free-roaming domestic dog densities to vary from 4–10 dogs/km^2^ in Ethiopia [35], 7.3 dogs/km^2^ in Chile [36], 76.8 dogs/km^2^ in Brazil [37], 113 dogs/km^2^ in villages in India [9], and all the way to 1081 dogs/km^2^ in Campeche, México [24] putting the density estimate found in Tulúm on the low to middle range of worldwide dog population estimates. Even in areas where dog densities are lower than it is in Tulúm, free-roaming dogs are negatively affecting humans and other species [35,36]. In Dhaka city, Bangladesh, where a comparable average dog density of 52 dogs/km^2^ was found, the dog population densities in different wards varied greatly, indicating that even within a city, dog populations should not be considered homogenous within a city [38]. Beyond describing the dog population, this has implications for dog population management, disease transmission, and risk analysis for different human populations [38].

The four transects with the highest population size per kilometer of transect length estimates were Transects 2, 3, 4, and 5. In addition to being in a more central part of the city, which could indicate higher human densities, these transects were the only four to go through areas known as “La Invasión”. “La Invasión” are areas of Tulúm where people have built houses on once vacant land. It is known for being an economically deprived area with dirt roads and less car traffic. In Transect 2, 75% of the dog encounters came from La Invasión, despite this area only representing 28.7% of the total Transect 2 length. In Transect 3, 72.7% of the dogs encountered came from the two areas of La Invasión despite only representing about 48% of the transect. A similar pattern is found in Transects 4 and 5 where 29.4% of dogs encountered were found in La Invasión section of Transect 4 while this area only represents 13.3% of the total Transect 4 length and 28.1% of dogs in Transect 5 were from La Invasión but this area only represents 15.2% of the total Transect 5 length. This pattern of dogs being overrepresented in areas of La Invasión when compared with the other areas of Transects 2, 3, 4, and 5 indicates that the population density of free-roaming dogs in La Invasión is likely greater than densities in other parts of the city. Higher free-roaming dog densities can be a function of higher human densities as was found in Zimbabwe [39] and in the Philippines [40]. However, whether La Invasión has higher human densities than other areas of Tulúm is unknown, but human density seems to be lower than central areas of the city where there are multi-story apartment buildings and houses are packed more tightly.

In addition, higher dog densities in La Invasión are likely related to the lower socioeconomic status of people living in this area. In Hermosilla, México, people of low socioeconomic status were four times more likely to report the presence of high dog densities in their neighborhoods than people from high socioeconomic status [41]. Similarly, in Buenos Aires, Argentina, lower income neighborhoods had higher dog densities than middle income neighborhoods [42]. With lower economic means, people may be more likely to keep dogs outside for convenience [43]. The dog population in La Invasión may also have a larger proportion of abandoned or stray dogs as people may be less likely to spay or neuter their animals and more likely to give up their pets when they do not have the resources to care for them. Additionally, when compared with other areas of Tulúm, La Invasión has much lower car traffic. Due to the high risk that cars pose to free-roaming dogs [44,45], owners may be less likely to let their dogs roam freely in areas of the city where car traffic is higher.

The lower dog populations per kilometer of transect length in transects 1, 6, and 7 when compared to Transects 2, 3, 4 and 5 are likely due to a combination of factors. The tourist influence in transects 1, 6, and 7 may be partly responsible for the lack of free-roaming dogs. For example, in Oaxaca, México, international tourists were more likely to be concerned over the welfare of free-roaming dogs, getting diseases from dogs, and feces on sidewalks and streets than both locals and in-country tourists [46]. These cultural differences in attitudes toward free- roaming dogs may be partially responsible for lower levels of free-roaming dogs living in transects where more American and European expats/tourists live. In addition to the cultural differences there may be in dog ownership between tourists and Mexican locals, tourists are less likely to have their pets with them or let their dogs roam free in a new area. Any dogs or puppies found in areas of heavy tourism may also have a greater chance of being “rescued” as tourists and expats are more likely to have the resources to rescue dogs and may be more likely to be concerned with the welfare of dogs [46]. This is evident by the existence of the expat/tourist run Tulúm Animal Rescue, which is involved in rescuing dogs and connecting rescuers (primarily tourists) to veterinarians and foster homes.

In addition to population size, the POPAN model predicts survival and capture probabilities. Due to low sample size and a low number of recaptures in the study, confidence intervals for survival and capture probabilities are large for all transects. Throughout the study area, survival probability was fairly high, above 73% for all transects and 95% or above for Transects 3, 4, 5, and 7. On the other hand, capture probabilities remained low for all transects (below 34%) except for Transect 2, which had an estimate capture probability of 65%. Interestingly, Transect 2 had both the lowest survival probability and the highest capture probability. A high survival rate and low capture rate could potentially be explained by the relationship between food availability and dog movement. In areas with high survival, there may be higher food availability meaning dogs living in these areas do not have to travel as far or as often for their food, making them less likely to cross transects and thus capture probabilities would be lower on these transects. The opposite of this could also be true, where areas with low survival probability, like Transect 2, may have lower food availability and thus dogs in this area may be crossing the transect more often, leading to a higher capture probability. While it would be interesting to compare survival and capture probabilities between transects, the differences in these values between transects are not significant due to large confidence intervals.

### Parasite prevalence and zoonotic implications

The four gastrointestinal parasites found during scat collection have varying impacts and prevalence in their canine hosts. Both *D*. *caninum* and *T*. *canis* infections tend to be mild in adult dogs, but *T*. *canis* can be deadly in puppies [47,48]. *Cystoisospora* spp. infections often cause diarrhea and sometimes more severe symptoms, such as bloody stool, vomiting, fever, and weight loss [49,50]. Finally, *Ancylostoma caninum* infections vary from asymptomatic cases to fatal cases of exsanguination and anemia [51]. As for prevalence, *D*. *caninum* was found in one fecal sample (4%); this low prevalence may be representative of the population or be due to low sample sizes. *D*. *caninum* rates previously reported in México vary widely from a low of 2.3% in a Yucatan town [52] to a high of 60% in Mérida [53]. Rates of *Cystoisospora* spp. and *T*. *canis* found in this study (8%) are consistent with those found around the world and in México. Fecal-flotation analysis of *Cystoisospora* spp. in dogs from the United States [54], Austria [55], and Argentina [56] found oocytes in 3–8.7% of samples, while *T*. *canis* was found in 5 to 13.3 percent of fecal samples from Mexicali [57], Yucatan [52], and México City [58]. The high prevalence of *A*. *caninum* (64%) was unsurprising given roughly similar rates of 62.5% and 73.8% in México City [58] and a Yucatan community [52], respectively.

Of the four gastrointestinal parasite species found during scat collection, *T*. *canis*, *D*. *caninum*, and *A*. *caninum* can infect humans with varying effects on human health. Human infection with *T*. *canis* can be asymptomatic or have a wide range of health impacts including fever, diarrhea, asthma, seizures, intestinal disorders, and rarely ocular larva migrans [59,60]. One study done in a Yucatan community found *T*. *canis* present in 12% of dog fecal samples analyzed and serological prevalence of *T*. *canis* in humans to be 29.2%, indicating that even when presence of active *T*. *canis* infections in dog populations is low, *T*. *canis* still poses a public health risk to human populations [47]. In contrast to *T*. *canis*, *D*. *caninum* infection in humans is rare and occurs as a result of accidental ingestion of dog fleas or louse [61]. *D*. *caninum* most commonly infects infants and children and causes mild diarrhea and abdominal pain [61]. Finally, *A*. *caninum* has two ways of infecting people, through ingestion, causing abdominal pain and diarrhea, and skin contact, leading to itchy lesions [62–65]. *A*. *caninum* has been implicated in a possible cause of an outbreak of eosinophilic enteritis in Queensland, Australia in 1988 [65], and *A*. *caninum* eggs were found in human feces for the first time in Brazil in 2020, indicating maturation and reproduction of *A*. *caninum* in humans is possible [62]. To prevent future outbreaks and infections of these zoonotic diseases in humans, more should be done to educate the public on the threat these parasites pose to people and veterinarians on practices that could reduce drug resistance.

## Conclusions

To decrease the spread of zoonotic diseases, special attention needs to be given to *A*. *caninum* as the most common parasite species found in dog feces and to people living in Tulúm’s “La Invasión” area where parasite prevalence and free-roaming dog populations may be higher. Given the lower socioeconomic status of people living in La Invasión, educating the public on the health threats free-roaming dogs pose cannot simply occur in veterinarian offices where lower income dog owners may be less likely to go. Targeted messaging is needed in these low-income areas to decrease the risk of zoonotic transmission of canine parasites. More global education on the threats free-roaming dogs pose to the public is also needed, as the free-roaming dog densities throughout Tulúm, México, and much of the world, are high.

## Supporting information

S1 Table POPAN models fitted for Transect 2. AICc values, Delta AICc values, AICc weights, model likelihoods, parameter count, and deviances for models fitted in MARK using POPAN function for Transect 2, ordered by lowest AICc (or highest AICc weights). A “t” corresponds to a parameter varying over time, meaning that models with *phi*(t), for example, had separate *phi* (survival) parameters between each visit to the transect. A “.” indicates that the given parameter was assumed to stay constant throughout the study period and thus for a model with *phi*(.), there would only be one *phi* parameter and thus only one survival rate estimated for the whole duration of the study. 4 of 8 possible models were able to be fit for this transect. Only one model, indicated with an asterisk, was used for estimating survival, capture probability, and population size based on AICc weights.
(DOCX)

S2 Table POPAN models fitted for Transect 3. AICc values, Delta AICc values, AICc weights, model likelihoods, parameter count, and deviances for all models fitted in MARK for Transect 3. 6 of 8 possible models were able to be fit for this transect. Model notation is described in S1 Table. Only one model, indicated with an asterisk, was used for estimating survival, capture probability, and population size based on AICc weights.
(DOCX)

S3 Table POPAN models fitted for Transect 4. AICc values, Delta AICc values, AICc weights, model likelihoods, parameter count, and deviances for all models fitted in MARK for Transect 4. 6 of 8 possible models were able to be fit for this transect. Model notation is described in S1 Table. Only one model, indicated with an asterisk, was used for estimating survival, capture probability, and population size based on AICc weights.
(DOCX)

S4 Table POPAN models fitted for Transect 5. AICc values, Delta AICc values, AICc weights, model likelihoods, parameter count, and deviances for all models fitted in MARK for Transect 5. 6 of 8 possible models were able to be fit for this transect. Model notation is described in S1 Table. Only one model, indicated with an asterisk, was used for estimating survival, capture probability, and population size based on AICc weights.
(DOCX)

S5 Table POPAN models fitted for Transect 6. AICc values, Delta AICc values, AICc weights, model likelihoods, parameter count, and deviances for all models fitted in MARK for Transect 6. 5 of 8 possible models were able to be fit for this transect. Model notation is described in S1 Table. Only one model, indicated with an asterisk, was used for estimating survival, capture probability, and population size based on AICc weights.
(DOCX)

S6 Table POPAN models fitted for Transect 7. AICc values, Delta AICc values, AICc weights, model likelihoods, parameter count, and deviances for all models fitted in MARK for Transect 7. 6 of 8 possible models were able to be fit for this transect. Model notation is described in S1 Table. Two models, indicated with asterisks, were used for calculating a weighted average of estimates for survival, capture probability, and population size.
(DOCX)

S1 Dataset Raw presence/absence data and variables for Lincoln-Peterson estimates for each transect. On Table 1, all sampling periods for each transect are indicated with basic dog detection information provided. On Table 2, the Lincoln-Peterson data are provided with values for M (number of individual dogs seen in first half of trap days), S (number of dogs seen in second half trap days), and R (number of dogs seen in the first and second half of trap days).
(XLSX)
