# Supplementary material for: Investigating the free-roaming dog population and gastrointestinal parasite diversity in Tulúm, México
Source: PLoS One. 2022 Oct 27;17(10):e0276880. doi: 10.1371/journal.pone.0276880 (PMC9612467; doi:10.1371/journal.pone.0276880)
Supplement: S1 Table — AICc values, Delta AICc values, AICc weights, model likelihoods, parameter count, and deviances for models fitted in MARK using POPAN function for Transect 2, ordered by lowest AICc (or highest AICc weights). A “t” corresponds to a parameter varying over time, meaning that models with phi(t), for example, had separate phi (survival) parameters between each visit to the transect. A “.” indicates that the given parameter was assumed to stay constant throughout the study period and thus for a model with phi(.), there would only be one phi parameter and thus only one survival rate estimated for the whole duration of the study. 4 of 8 possible models were able to be fit for this transect. Only one model, indicated with an asterisk, was used for estimating survival, capture probability, and population size based on AICc weights. (DOCX) [file pone.0276880.s001.docx]

**S1 Table. POPAN models fitted for Transect 2.**

| **Model** | **AICc** | **Delta AICc** | **AICc Weights** | **Model Likelihood** | **Num. Parameter** | **Deviance** |
| --- | --- | --- | --- | --- | --- | --- |
| *phi*(.)*p*(.)*pent*(t)* | 80.7469 | 0.0000 | 1.0000 | 1.0000 | 9 | -17.0865 |
| *phi*(t)*p*(.)*pent*(t) | 186.7551 | 106.0082 | 0.0000 | 0.0000 | 15 | -25.0782 |
| *phi*(.)*p*(t)*pent*(t) | 250.8621 | 170.1152 | 0.0000 | 0.0000 | 16 | -24.3047 |
| *phi*(t)*p*(t)*pent*(t) | 374.2070 | 293.4601 | 0.0000 | 0.0000 | 17 | -27.6264 |

AICc values, Delta AICc values, AICc weights, model likelihoods, parameter count, and deviances for models fitted in MARK using POPAN function for Transect 2, ordered by lowest AICc (or highest AICc weights). A “t” corresponds to a parameter varying over time, meaning that models with *phi*(t), for example, had separate *phi* (survival) parameters between each visit to the transect. A “.” indicates that the given parameter was assumed to stay constant throughout the study period and thus for a model with *phi*(.), there would only be one *phi* parameter and thus only one survival rate estimated for the whole duration of the study. 4 of 8 possible models were able to be fit for this transect. Only one model, indicated with an asterisk, was used for estimating survival, capture probability, and population size based on AICc weights.
