# Supplementary material for: Investigating the free-roaming dog population and gastrointestinal parasite diversity in Tulúm, México
Source: PLoS One. 2022 Oct 27;17(10):e0276880. doi: 10.1371/journal.pone.0276880 (PMC9612467; doi:10.1371/journal.pone.0276880)
Supplement: S4 Table — AICc values, Delta AICc values, AICc weights, model likelihoods, parameter count, and deviances for all models fitted in MARK for Transect 5. 6 of 8 possible models were able to be fit for this transect. Model notation is described in S1 Table. Only one model, indicated with an asterisk, was used for estimating survival, capture probability, and population size based on AICc weights. (DOCX) [file pone.0276880.s004.docx]

**S4 Table. POPAN models fitted for Transect 5.**

| **Model** | **AICc** | **Delta AICc** | **AICc Weights** | **Model Likelihood** | **Num. Parameter** | **Deviance** | |
| --- | --- | --- | --- | --- | --- | --- | --- |
| *phi*(.)*p*(.)*pent*(t)* | 110.7482 | 0.0000 | 0.99994 | 1.0000 | 4 | -58.1258 |  |
| *phi*(t)*p*(.)*pent*(t) | 131.5366 | 20.7884 | 0.00003 | 0.0000 | 13 | -66.4451 |  |
| *phi*(.)*p*(t)*pent*(t) | 131.9789 | 21.2307 | 0.00002 | 0.0000 | 13 | -66.0028 |  |
| *phi*(t)*p*(t)*pent*(t) | 157.2630 | 46.5148 | 0.00000 | 0.0000 | 19 | -72.2521 |  |
| *phi*(t)*p*(.)*pent*(.) | 12805.466 | 12694.7181 | 0.00000 | 0.0000 | 8 | 12785.352 |  |
| *phi*(.)*p*(.)*pent*(.) | 12827.907 | 12717.1588 | 0.00000 | 0.0000 | 3 | 12821.307 |  |

AICc values, Delta AICc values, AICc weights, model likelihoods, parameter count, and deviances for all models fitted in MARK for Transect 5. 6 of 8 possible models were able to be fit for this transect. Model notation is described in S1 Table. Only one model, indicated with an asterisk, was used for estimating survival, capture probability, and population size based on AICc weights.
