# Supplementary material for: Investigating the free-roaming dog population and gastrointestinal parasite diversity in Tulúm, México
Source: PLoS One. 2022 Oct 27;17(10):e0276880. doi: 10.1371/journal.pone.0276880 (PMC9612467; doi:10.1371/journal.pone.0276880)
Supplement: S6 Table — AICc values, Delta AICc values, AICc weights, model likelihoods, parameter count, and deviances for all models fitted in MARK for Transect 7. 6 of 8 possible models were able to be fit for this transect. Model notation is described in S1 Table. Two models, indicated with asterisks, were used for calculating a weighted average of estimates for survival, capture probability, and population size. (DOCX) [file pone.0276880.s006.docx]

**S6 Table. POPAN models fitted for Transect 7.**

| **Model** | **AICc** | **Delta AICc** | **AICc Weights** | **Model Likelihood** | **Num. Parameter** | **Deviance** |
| --- | --- | --- | --- | --- | --- | --- |
| *phi*(.)*p*(.)*pent*(t) | 80.4848 | 0.0000 | 0.83161 | 1.0000 | 7 | 3.9649 |
| *phi*(t)*p*(t)*pent*(t) | 83.6789 | 3.1941 | 0.16839 | 0.2025 | 17 | -1.6411 |
| *phi*(.)*p*(t)*pent*(t) | 170.2225 | 89.7377 | 0.00000 | 0.0000 | 13 | 1.9025 |
| *phi*(t)*p*(.)*pent*(t) | 219.6905 | 139.2057 | 0.00000 | 0.0000 | 14 | 0.3705 |
| *phi*(.)*p*(.)*pent*(.) | 2871.2897 | 2790.8049 | 0.00000 | 0.0000 | 2 | 2815.1697 |
| *phi*(t)*p*(.)*pent*(.) | 2880.1187 | 2799.6339 | 0.00000 | 0.0000 | 8 | 2848.1187 |

AICc values, Delta AICc values, AICc weights, model likelihoods, parameter count, and deviances for all models fitted in MARK for Transect 7. 6 of 8 possible models were able to be fit for this transect. Model notation is described in S1 Table. Two models, indicated with asterisks, were used for calculating a weighted average of estimates for survival, capture probability, and population size.
